# A systematic review on bioactive glass as a treatment for limb osteomyelitis and infected nonunion

**DOI:** 10.1302/2633-1462.610.BJO-2025-0084.R2

**Published:** 2025-10-13

**Authors:** Henry A. Claireaux, Helen S. M. Smith, Andrew M. Edwards, Julian R. Jones, Arul Ramasamy

**Affiliations:** 1 Department of Materials, Imperial College London, London, UK; 2 Academic Department of Military Trauma & Orthopaedics, Birmingham, UK; 3 Centre for Bacterial Resistance Biology, Imperial College London, London, UK; 4 Centre for Injury Studies, Imperial College London, London, UK

**Keywords:** Biomaterials, Bioactive glass, Osteomyelitis, Infected nonunion, Infection, bone infection, nonunions, randomized controlled trials, infections, antibiotics, calcium sulphate, patient-reported outcome measures (PROMs)

## Abstract

**Aims:**

Osteomyelitis and infected nonunion cause devastating morbidity and are difficult to treat. Antimicrobial resistance further complicates musculoskeletal infection and is a significant global problem, including in low- and middle-income countries. Extensively drug-resistant bacteria and high rates of musculoskeletal infection have been identified during the Russian-Ukrainian war. Biomaterials with antimicrobial properties unrelated to antibiotics represent a potential solution. Bioactive glass, for example, has shown promise as a bone void filler. It binds to bone, stimulates bone formation, causes no harmful immune response, and has antimicrobial properties. This systematic review appraises the evidence for bioactive glass as a treatment for osteomyelitis and infected nonunion.

**Methods:**

A comprehensive search of MEDLINE and EMBASE was performed with research librarian guidance. Articles were screened and assessed for risk of bias by two blinded authors. No date limitations were used. Methodology was guided by the Cochrane Handbook and the PRISMA statement. Data were compiled and narratively synthesized.

**Results:**

We included 24 observational studies on 957 patients. Reported outcomes were heterogeneous, with patient-reported outcome measures available in only one study. Most studies were small and at considerable risk of bias. Studies supported bioactive glass use with high rates of bone healing and infection resolution. Comparative studies found non-inferiority with established treatments such as antibiotic-containing calcium sulphate and polymethylmethacrylate cement spacers. Few significant bioactive glass-related complications were reported.

**Conclusion:**

This review demonstrates the potential of bioactive glass as a treatment for osteomyelitis and infected nonunion. Widespread uptake over established treatments is likely to require further supporting evidence, such as high-quality randomized controlled trials, to understand the role of biomaterials in treating these challenging conditions. Future work should examine 3D-printed bioactive glass hybrids, which may have biomechanical advantages for large bone defects.

Cite this article: *Bone Jt Open* 2025;6(10):1248–1259.

## Introduction

Fracture-related infections and osteomyelitis present significant challenges. Open fractures are common, with 1,175 patients included in a three-month UK observational study at 51 sites.^1^ Bone infection is a major complication of open fractures, and this occurs in around 20% of open tibial fractures.^2,3^ Infection may impede fracture healing, leading to nonunion, which can cause a poorer quality of life than myocardial infarction, stroke, and some cancers.^4^

Treating nonunions is expensive for health systems, with direct costs of £29,204 reported in the UK.^5^ A German study estimated a median total socioeconomic burden of €123,334, including €85,714 of lost productivity.^6^ Osteomyelitis treatment and perioperative care are often challenging due to high rates of medical comorbidity in this patient group.^7-9^ Limited antibiotic penetration into bone means the infection can persist despite appropriate treatment. The mean length of stay for patients with osteomyelitis is 17.5 days, with a 20% risk of rehospitalization after discharge.^10^ Improving the care for fracture-related infection was identified as a patient and clinician priority in a recent consultation.^11^

Bone infections are notoriously difficult to treat, frequently involving debridement of non-viable bone, microbiological sampling, and implantation of local antibiotics. These may be powdered, in a soluble carrier (e.g. calcium sulphate), or in polymethylmethacrylate (PMMA) cement beads.^12^ Second-stage operations may follow to remove temporary spacers and address bone defects. Patients are treated with intravenous antibiotics, necessitating hospital admission, daily clinic attendance, or home visits. Antimicrobial resistance further complicates musculoskeletal infection and is a significant global problem, including in low- and middle-income countries.^13^ Extensively drug-resistant bacteria and high rates of musculoskeletal infection have been identified during the Russian-Ukrainian war.^14-16^ Incomplete bacterial killing in an osteomyelitic focus may also select for resistance, as only susceptible bacteria are killed. Biomaterials with antimicrobial effects may improve outcomes in bone infection by reducing systemic antibiotic side effects and killing bacteria locally, including strains resistant to antibiotics.

**Fig. 1:**
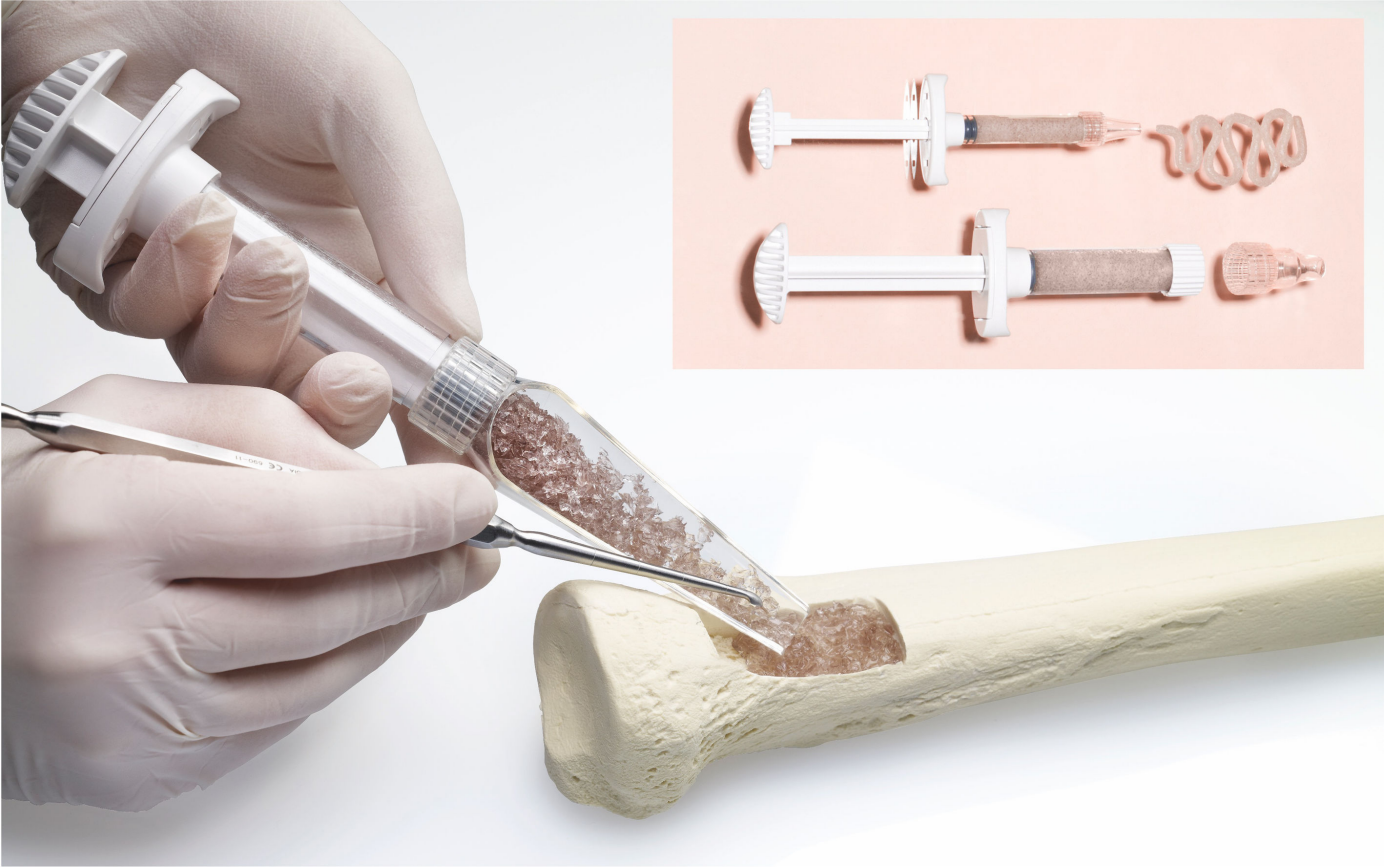
S53P4 Bonalive bioactive glass granules courtesy of Bonalive Biomaterials (Finland).

**Fig. 2:**
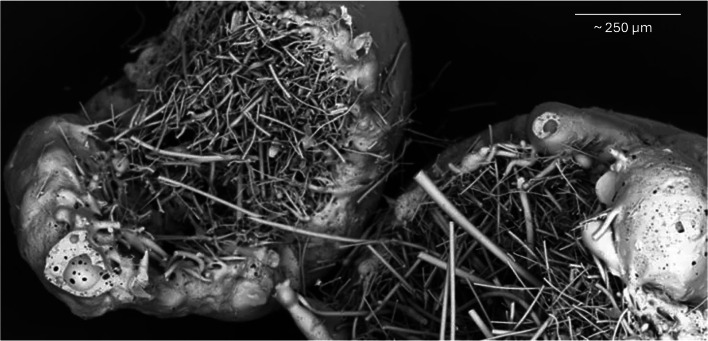
45S5 FIBERGRAFT bioactive glass granules (DePuy Synthes, USA), courtesy of Prosidyan (USA).

Biomaterials, including bioactive glass, have shown promise as synthetic bone grafts in orthopaedic surgery due to dual biological properties of osteoinduction and antimicrobial action.^17-20^ S53P4 (Bonalive, Finland) and 45S5 glasses (Fibergraft, De Puy Synthes, USA; Novabone, USA; Glassbone, Noraker, France) are commercially available and licensed for clinical use (Figure 1 and Figure 2). Bioactive glass is commonly supplied either as granules (0.5-2mm) or a putty, in which particles are suspended in a glycerol carrier. Fibergraft is different, in that larger granules are filled with glass fibres. Common orthopaedic indications are filling bone defects, including after debridement for osteomyelitis. Bioactive glass also has several applications within otorhinolaryngology, dentistry, and spinal surgery.

**Fig. 3:**
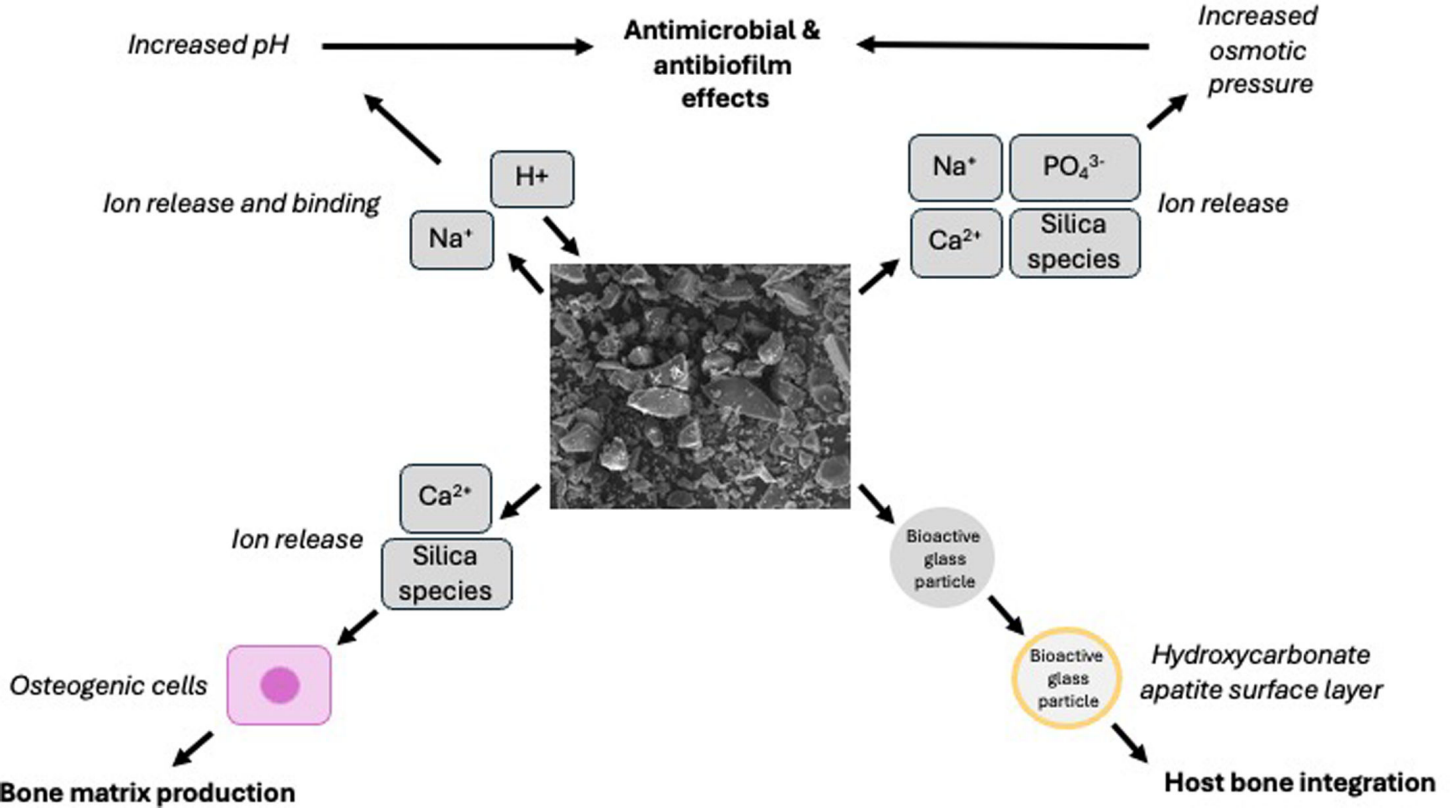
Bioactive glass mechanism of action.

Using bioactive glass as a bone void filler avoids the donor site morbidity encountered with autologous bone grafting. As bioactive glass biodegrades, ions are released into surrounding tissues, causing an increase in osmotic pressure and pH, contributing to an antimicrobial effect against osteomyelitis and biofilm (Figure 3).^19^ Bioactive glass dissolution also causes a hydroxycarbonate apatite layer to form on the surface of the glass, which integrates strongly with host bone.^18,21^ Silica species and calcium ions stimulate osteogenic cells to form new bone matrix (Figure 3).^18,22^ No harmful immune response occurs, and there is no host toxicity.^18,23^

The indications and adverse events associated with bioactive glass use in orthopaedics are not fully understood. This systematic review aims to appraise the evidence for bioactive glass as a treatment for osteomyelitis and infected nonunion.

## Methods

This systematic review was conducted following Cochrane Handbook guidelines, and reporting is as per the PRISMA statement.^24,25^ The protocol was developed prospectively and registered on PROSPERO (CRD42025646354).^26^

### Search strategy

A comprehensive search was designed with research librarian guidance (Supplementary Material). This was run on MEDLINE (1946 to present) and EMBASE (1974 to present) on 5 June 2025. No date or language limitations were applied.

### Study selection

**Fig. 4:**
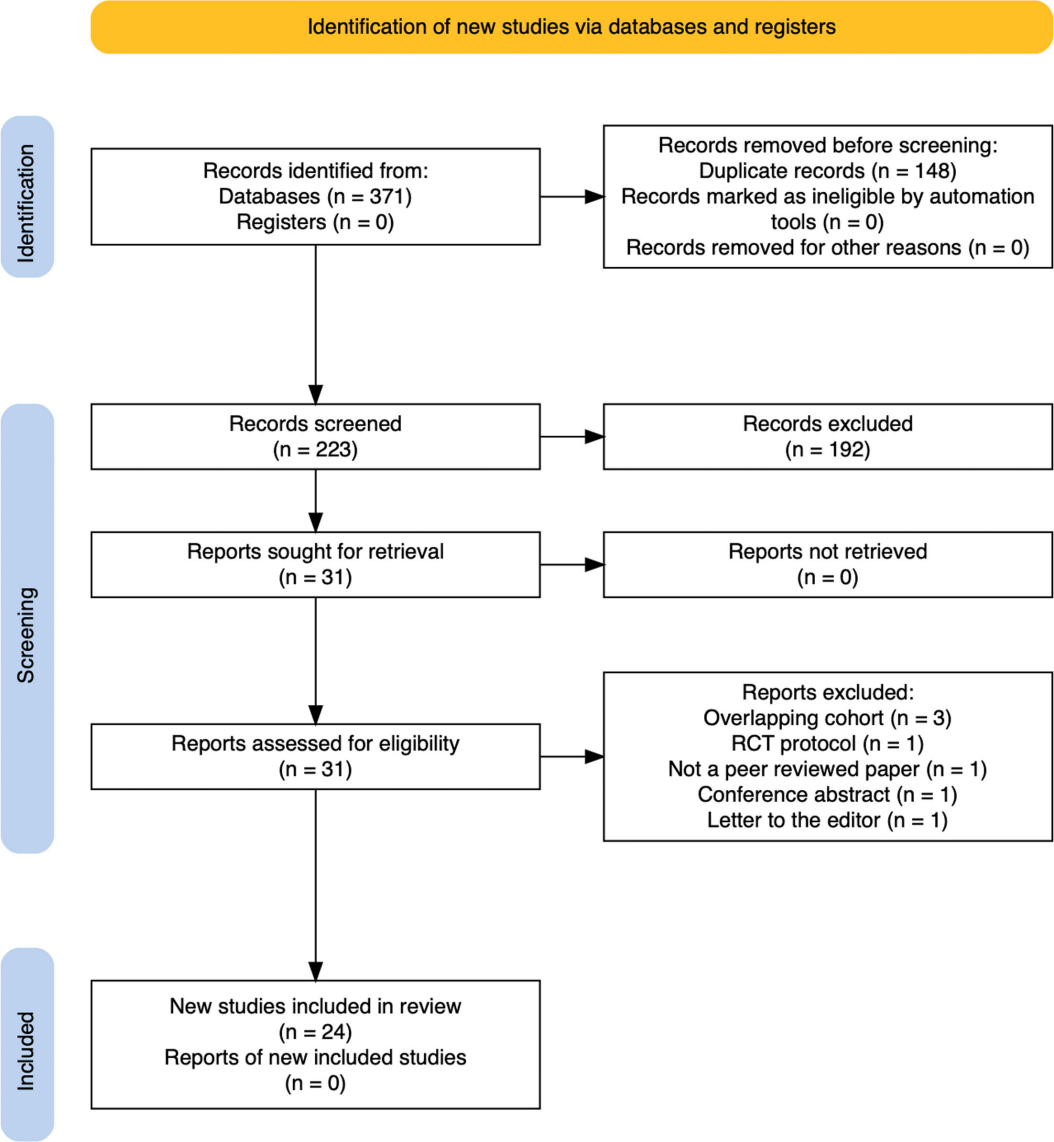
PRISMA diagram. RCT, randomized controlled trial.

Search results were merged and deduplicated before blinded abstract screening by HAC and HSMS using Rayyan (Rayyan, USA).^27^ Full texts were screened in duplicate according to predefined criteria (Figure 4). Disagreements were discussed and referred to AR if consensus could not be reached.

Studies including patients treated with bioactive glass for osteomyelitis, fracture-related infection, and infected nonunion were included. Case reports, letters, conference abstracts, and animal studies were excluded. Where studies reported overlapping cohorts from one institution, the most comprehensive was included. Observational studies were categorized as cohort or case series using the definition from Dekkers et al.^28^

Primary outcomes were fracture healing and infection clearance. Secondary outcomes were patient-reported outcome measures (PROMs) and adverse events. Citations were managed in Zotero (Zotero, USA).^29^

### Patient characteristics

Cumulatively, the included studies had 957 participants. Study size ranged from 3 to 132 patients. Overall, 277 of 957 (29%) of participants were female. The mean age was 46.6 years (SD 12.4). The indication for bioactive glass was osteomyelitis in 17 studies and infected nonunion in the remaining seven (Table I).

### Data extraction and statistical analysis

Data were extracted and risk of bias was assessed in duplicate by HC and HS. Risk of bias assessment was blinded and used the National Institutes of Health (NIH) tool.^30^ The heterogeneous nature of the included observational studies precluded meta-analysis.

## Results

### Study selection

Database searching identified 223 unique abstracts, 31 full texts were reviewed, and 24 observational studies were included (Figure 4).^31-54^ One randomized controlled trial (RCT) was identified that has not yet been reported.^55^ The corresponding author was contacted, and data collection is ongoing.

### Study characteristics

Seven comparative cohort studies, 15 non-comparative cohort studies, and two case series were included (Table I). Six collected data prospectively. Studies were conducted between 2007 and 2022.

**Table I.** Characteristics of included studies.

Study	Indication	Study design and level of evidence	Type of bioactive glass used	Comparitor (if any)	Outcome measures	Key findings	Adverse events
Lindfors et al, 2010^31^ Finland	Osteomyelitis	Cohort study (retrospective) (level 3)	S53P4 granules (Bonalive, Finland)	None	Bony union	9 patients healed without complications	1 superficial wound infection due to flap failure. 1 infection due to a deep haematoma. No bioactive glass-related complications.
Drago et al, 2013^32^ Italy	Osteomyelitis	Cohort study (prospective) (level 3)	S53P4 granules (Bonalive, Finland)	None	Infection clearance	No sign of infection in 88.9%. 2 patients had infection recurrence.	1 serum wound leak. 1 reoperation.
McAndrew et al, 2013^33^ Ireland	Osteomyelitis	Case series (retrospective) (level 4)	S53P4 bioactive glass (Bonalive, Finland)	None	Functional, antibiotic requirement, clearance of infection, biomaterial integration	Pain ceased, function returned, all antibiotics stopped, no radiological evidence of osteomyelitis, glass has integrated with bone on imaging	None reported
Romanò et al, 2014^34^ Italy	Osteomyelitis	Cohort study (retrospective and comparative) (level 3)	S53P4 granules (Bonalive, Finland)	Antibiotic-loaded hydroxyapatite and calcium sulphate compound (PerOssal, Aap Biomaterials, Germany). 2- Mixture of tricalcium phosphate (Calcibon, Biomet, Germany) and antibiotic-loaded demineralized bone matrix (Targobone, Germany)	Infection clearance	Infection controlled in 25/27 bioactive glass group, vs 24/27 antibiotic-loaded hydroxyapatite and calcium sulphate group, vs 19/22 tricalcium phosphate and an antibiotic-loaded demineralized bone matrix group. Reduced wound complications in bioactive glass group.	Prolonged serum leakage: 1/27 bioglass group, 7/27 antibiotic-loaded hydroxyapatite and calcium sulphate group, 6/22 antibiotic-loaded demineralized bone matrix group
Ferrando et al, 2017^35^ Spain	Osteomyelitis	Cohort study (retrospective and comparative) (level 3)	S53P4 bioactive glass (Bonalive, Finland)	Calcium sulphate antibiotic beads (Stimulan)	Infection clearance	1/12 recurrence of infection in each group. No difference in complication rate.	Seroma, delayed wound healing, haematoma
Lindfors et al, 2017^36^ Europe + Azerbaijan	Osteomyelitis	Cohort study (retrospective) (level 3)	S53P4 granules (Bonalive, Finland)	None	Grading system	90% treatment success. ‘Poor' outcome graded in 5% of patients in 1 stage group vs 33% in 2 stage group	8% persistence of infection
Malat et al, 2018^37^ Germany	Osteomyelitis and infected nonunion	Cohort study (retrospective) (level 3)	S53P4 granules (Bonalive, Finland)	None	Bony union, weightbearing	Bone healing in 26/37 (70.3 %) at 6 months and 35/42 (83.3%) at 12 months. 40 patients (80%) have achieved full weightbearing after a mean of 4 months.	24% had adverse events including implant failure, soft-tissue defect requiring flap, persistent infection. No local reaction to bioactive glass.
Gaiarsa et al, 2019^38^ Brazil	Infected nonunion	Cohort study (retrospective) (level 3)	S53P4 granules (Bonalive, Finland)	None	Bony union, RUST score^56^	Radiological healing in 17/18 at 2 years with mean RUST score of 10.2	None reported
Geurts et al, 2019^39^ Netherlands	Osteomyelitis	Cohort study (retrospective and comparative) (level 3)	S53P4 bioactive glass (Bonalive, Finland)	Two-stage using gentamicin-loaded PMMA beads	Cost, infection clearance	Lower cost in bioactive glass group (€20,568.31 vs €27,131.69). Infection eradication higher in bioactive glass group (92% vs 80%).	No significant differences in adverse events between groups. 1 femoral fracture in bioactive glass group. 1 death and 1 amputation in control group.
Iacopi et al, 2020^40^ Italy	Osteomyelitis (diabetic foot)	Cohort study (prospective) (level 3)	S53P4 granules and putty (Bonalive, Finland)	None	Bony union	80% healed in mean time of 34 (SD 2) days	1 reoperation
Oosthuysen et al, 2020^41^ South Africa	Osteomyelitis	Cohort study (retrospective) (level 3)	S53P4 granules (Bonalive, Finland)	None	Symptom resolution	22/24 complete resolution of symptoms	1 serous wound discharge that resolved with local wound care
Testa et al, 2020^42^ Italy	Infected nonunion	Case series (retrospective) (level 4)	S53P4 granules (Bonalive, Finland)	None	Bony union, AOFAS score,^57^ ASAMI score^58^	30% of cases treated with bioactive glass. No significant diﬀerences in AOFAS and ASAMI scores were found between bioactive glass usage and non-usage. All bioactive glass patients had excellent or good radiological results.	No comparison between groups reported. Pin site infections, K-wire breakage, screw breakage occurred.
De Giglio et al, 2021^43^ Italy	Osteomyelitis (diabetic foot)	Cohort study (retrospective and comparative) (level 3)	S53P4 granules and putty (Bonalive, Finland)	Debridement with oscillating saw leaving a void, primary closure. Antibiotics given.	Infection clearance, antibiotic requirement	Osteomyelitis resolution higher in bioactive glass group (90% vs 62%). Bioactive glass group had 81% lower antibiotic requirement.	No reoperations or systemic complications in bioactive glass group
Kastrin et al, 2021^44^ Slovenia	Osteomyelitis (diabetic foot, with septic joint)	Cohort study (retrospective and comparative) (level 3)	S53P4 bioactive glass mixed with venous blood (no manufacturer stated)	Septopal Chain gentamicin beads (Zimmer Biomet, Germany)	Antibiotic requirement, reoperation	No additional antibiotics or reoperations in bioactive glass group. 3 patients in gentamicin bead group required further intervention including amputation.	None in bioactive glass group. Valgus deformity in gentamicin bead group.
Kojima et al, 2021^45^ Brazil	Osteomyelitis	Cohort study (prospective) (level 3)	S53P4 granules and putty (Bonalive, Finland)	None	Bony union, DASH^59^ and Lysholm scores	No reoperation or wound problems. Partial incorporation and adequate bone formation on imaging. Satisfactory functional scores.	2 wound serum leak which resolved without intervention
Rodríguez et al, 2021^46^ Spain	Osteomyelitis (diabetic foot)	Cohort study (prospective) (level 3)	S53P4 granules and putty (Bonalive, Finland)	None	Infection clearance, bony union, biomaterial osseointegration	0/6 persistent infection, 4/6 healing rate, 4/6 complete bioactive glass osseointegration at 2 years	1 new osteomyelitis focus requiring amputation, 1 flap failure requiring amputation, 1 wound dehiscence
Steinhausen et al, 2021^47^ Germany	Osteomyelitis and infected nonunion	Cohort study (retrospective and comparative) (level 3)	S53P4 bioactive glass (Bonalive, Finland)	Autologous bone graft	Infection clearance, weightbearing, bony union	No significant difference between groups. Reinfection 29% in bioactive glass vs 9% in bone graft. Full weightbearing 92% in bioactive glass vs 97% in bone graft. Bone healing 77% in bioactive glass vs 78% in bone graft.	43% complication rate in bioactive glass vs 63% in bone graft. Reinfection higher in bioactive glass but more reoperations in bone graft group. Donor site morbidity seen in bone graft group.
Van Vugt et al, 2021^48^ Netherlands	Infected nonunion	Cohort study (retrospective) (level 3)	S53P4 bioactive glass (Bonalive, Finland)	None	Consolidation, weightbearing, infection clearance, RUST score	All had clinical consolidation, pain free full weightbearing, complete eradication of infection, mean RUST score 7.8	1 screw breakage, 1 persistent fistula requiring reoperation, 1 slow healing requiring reoperation with BMAC injection
Van Vugt et al, 2021^49^ Netherlands	Osteomyelitis	Cohort study (prospective) (level 3)	S53P4 bioactive glass (Bonalive, Finland)	None	Infection clearance	85% infection eradication, 89% infection free at 1 year	3 fractures through bone window, 1 of whom died after pulmonary embolus following this fracture. 1 failure of fixation without reinfection. 6 minor wound problems related to persisting/early recurrent infection.
Aurégan et al, 2022^50^ Europe	Osteomyelitis and infected nonunion	Cohort study (retrospective) (level 3)	S53P4 granules (Bonalive, Finland)	None	Bony union, infection clearance	Bony union in 7/8. No recurrence of infection	None directly related to bioactive glass are reported
Epstein and Ferreira, 2022^51^ South Africa	Osteomyelitis	Cohort study (retrospective and comparative) (level 3)	S53P4 bioactive glass (Bonalive, Finland)	Antibiotic-loaded cement spacers. 2- Modified Lautenbach irrigation systems. 3- Antibiotic-impregnated bone substitutes (Cerament G/V or Osteset).	Infection clearance	97% remission of infection in bioactive glass group (n = 35) vs 97% in irrigation, 82% in cement spacer, 90% in Cerament G/V, 89% in Osteoset	Not reported
Lazzeri et al, 2022^52^ Italy	Osteomyelitis	Cohort study (prospective) (level 3)	S53P4 granules and putty (Bonalive, Finland)	None	Bony union	All achieved healing at latest follow-up, no reoperations, radiological progression to normal anatomy	None reported
Dell'Aquila et al, 2023^53^ Brazil	Osteomyelitis	Cohort study (retrospective) (level 3)	S53P4 granules (Bonalive, Finland)	S53P4 putty / granules and putty	Infection clearance	Infection was eradicated in 85.9% at 6 months and 87.2% at 12 months	Not reported
Gatti et al, 2024^54^ Italy	Osteomyelitis and infected nonunion	Cohort study (retrospective) (level 3)	S53P4 granules ± putty (Bonalive, Finland)	None	Infection clearance, bony union	Osteomyelitis resolved in 22/24 (minimum 1 year follow-up). Nonunion healing in 11/14 (mean healing time 9.1 (SD 4.9) months).	Serous discharge and wound dehiscence

AOFAS, American Orthopaedic Foot and Ankle Society; ASAMI, Association for the Study and Application of Methods of Ilizarov; BMAC, bone marrow aspirate concentration; DASH, Disabilities of the Arm, Shoulder and Hand questionnaire; PMMA, polymethylmethacrylate; RUST, Radiographic Union Scale in Tibial fractures.

### Quality of included studies

All included studies were observational (level III to IV evidence) and are inherently at high risk of bias.^60^Table II shows the NIH quality rating for comparative cohort studies; these commonly lacked sample-size calculations and blinding of outcome assessors. Case series commonly lacked consecutive recruitment and detailed outcome reporting.

**Table II.** National Institutes of Health (NIH) quality assessment of comparative cohort studies.

Study	NIH quality assessment	Objective clearly stated	Population clearly defined	Participation rate 50% of eligible	Clear inclusion and exclusion	Sample size justification	Exposure prior to outcome measurement	Adequate follow-up time	Exposures clearly defined and consistently applied	Outcome measures clearly defined and implemented consistently	Blinding of outcome assessors	Loss to follow-up 20% or less	Confounders considered and adjusted for statistically
Romanò et al, 2014^34^	Fair	Good	Good	Good	Good	Poor	Good	Good	Good	Good	Poor	Good	Good
Ferrando et al, 2017^35^	Fair	Good	Good	Good	Good	Poor	Good	Good	Good	Good	Poor	Good	Good
Geurts et al, 2019^39^	Fair	Good	Good	Good	Good	Poor	Good	Good	Good	Good	Poor	Good	Good
De Giglio et al, 2021^43^	Fair	Good	Good	Good	Good	Poor	Good	Good	Good	Good	Poor	Good	Good
Kastrin et al, 2021^44^	Fair	Good	Good	Good	Good	Poor	Good	Good	Good	Good	Poor	Good	Good
Steinhausen et al, 2021^47^	Fair	Good	Good	Good	Good	Fair	Good	Good	Good	Good	Poor	Good	Good
Epstein et al, 2022^51^	Poor	Good	Good	Good	Good	Poor	Good	Good	Good	Good	Poor	Good	Poor

### Interventions

All studies used S53P4 bioactive glass granules and/or putty (Bonalive, Finland). Comparison groups in cohort studies included antibiotic-containing PMMA cement beads, antibiotic-containing calcium sulphate beads, autologous bone graft, irrigation systems, and leaving a bone void. All studies involved systemic antibiotic administration. Published studies on 45S5 Novabone (Novabone), 45S5 Fibergraft (De Puy Synthes), and Glassbone (Noraker) did not meet the inclusion criteria, for example, because they related to spinal surgery.

### Outcome measures

Outcomes were heterogeneously reported using many outcome measurement instruments and timepoints (Table I). Only Kojima et al^45^ reported PROMs, i.e. the Disabilities of the Arm, Shoulder and Hand questionnaire (DASH)^59^ and Lysholm scores.^61^ The most reported outcomes were resolution of infection and bony healing.

### Adverse events

Adverse events were reported in 19 studies, with five reporting no adverse events in patients treated with bioactive glass (Table I). No studies reported significant differences in adverse events between bioactive glass and comparison treatments. Persistence of infection and wound problems (e.g. serum leak) were commonly reported.

### Outcomes

A total of 17 studies on 729 patients examined osteomyelitis. Successful bone healing, infection clearance, and cost were reported (Table I). Romanò et al^34^ reported that S53P4 is not inferior in clearing infection to antibiotic-loaded calcium sulphate or antibiotic-loaded tricalcium phosphate and demineralized bone matrix. Geurts et al^39^ reported greater infection clearance and lower cost in one-stage S53P4 treatment compared to two stages with PMMA beads. De Giglio et al^43^ reported 90% infection clearance with S53P4 compared to 62% when the bone void was not filled.

Seven studies on 228 patients examined infected nonunion. Successful bone healing, clearance of infection, and good functional outcome scores were reported (Table I).

## Discussion

This systematic review suggests that bioactive glass may have an important role in orthopaedics, in particular for difficult-to-treat cases of osteomyelitis and infected nonunion that are refractory to other treatments. For example, many patients who were successfully treated with S53P4 had not been responding to antibiotics for several months. It should be noted that all included studies concurrently treated patients with systemic antibiotics. In the context of increasing antimicrobial resistance, it is important to understand the role of biomaterials with local antimicrobial effects independent of antibiotic medicines.

Studies comparing bioactive glass to established treatments for osteomyelitis and infected nonunion reported non-inferiority and acceptable adverse event profiles. However, there was significant heterogeneity in study design, operative technique, and outcome reporting. All included studies were observational, unblinded, and at risk of selection and reporting biases. No studies justified sample sizes, meaning they may be underpowered to detect clinically important differences between groups.

There was no consensus on outcome reporting, and only one study reported PROMs. A systematic review of outcome reporting in surgically treated lower limb osteomyelitis showed that recurrence of infection and pain were the most reported outcomes.^62^ Core outcome sets for osteomyelitis and infected nonunion would standardize reporting and allow comparison between studies, including pooled analysis for each condition.

No RCTs have compared bioactive glass with established treatments for osteomyelitis or infected nonunion. National registries, such as the UK Bone and Joint Infection Registry (BAJIR), can provide useful observational data to guide orthopaedic decision-making.^63^ Large sample sizes and rigorous study design may limit the biases inherent in observational research. However, this approach relies on sufficient numbers of patients receiving the interventions of interest. High-quality RCTs comparing bioactive glass to established treatments are required to improve our understanding of this biomaterial. For example, a RCT comparing S53P4 bioactive glass to antibiotic-containing hydroxyapatite/calcium sulphate (Cerament; Bonesupport, Sweden) after debridement of osteomyelitis would provide information on clinical and cost-effectiveness to guide decision-making. Interventional research in bone infection and limb reconstruction is limited by challenges in recruiting adequate numbers of patients when compared to common conditions such as distal radius fracture or elective hip arthroplasty for osteoarthritis.

Bioactive glass is commercially available as granules or putty. Granules are brittle and will break under loading, such as in weightbearing. Putty cannot bear a load, and the glycerol carrier can inhibit the granules’ bioactivity. Next-generation biomaterials such as 3D-printed ‘Bouncy Bioglass’ hybrids aim to additionally allow cyclic loading.^64^ Studies comparing commercially available bioactive glass with established treatments will pave the way for trials on emerging biomaterials to drive translation from bench to bedside.

This is the largest and most comprehensive systematic review to date.^65-70^ Significant limitations are inherent in the included studies, which are all observational and at high risk of bias. Osteomyelitis and infected nonunion are distinct conditions in terms of diagnosis, infection behaviour, treatment, and outcome measurement. Relatively few patients in included studies were treated for infected nonunion (228 of 957). Conclusions from this review should be applied with consideration of each specific diagnosis. Reference lists were checked for additional studies, but it is possible that relevant published work may not have been identified.

In conclusion, this review demonstrates the potential of bioactive glass as a treatment for osteomyelitis and infected nonunion. Widespread uptake over established treatments is likely to require further supporting evidence, such as high-quality RCTs, to understand the role of biomaterials in treating these challenging conditions.

**Take home message**

- Studies supported bioactive glass use with high rates of bone healing and infection resolution.

- Comparative studies found non-inferiority with established treatments such as antibiotic-containing calcium sulphate and polymethylmethacrylate cement spacers.

- Few significant bioactive glass-related complications were reported.

- This review demonstrates the potential of bioactive glass as a treatment for osteomyelitis and infected nonunion.

- Future work should examine 3D-printed bioactive glass hybrids, which may have biomechanical advantages for large bone defects.

## Data Availability

All data generated or analyzed during this study are included in the published article and/or in the supplementary material.
